# The Story of the Insane from Year to Year

**Published:** 1900-04-21

**Authors:** 


### THE STORY OF THE INSANE FROM YEAR TO YEAR.

Twelfth Series.

### THE ROYAL ASYLUM, DUNDEE.

At this asylum the numbers fell during the year 1898 from 430 to 418. There were 118 first admission, 24 readmissions, 60 recoveries, 51 discharged relieved, and 38 deaths. The recoveries stand at 42·25 per cent. of the admissions, and the deaths at 6 64 per cent. of the average number resident. Cerebral and spinal diseases are responsible for 15 of the deaths, consumption for 3, pneumonia for 1, and gangrene of the lung for 1. Of the year's admissions 52 owed their insanity to such moral causes as domestic trouble, mental anxiety, religion, &c. Intemperance in drink was the cause in 37 instances, and hereditary influence in 17. Of the total number of patients, 83 were of the private class; and this principle of treating private and pauper cases in the same asylum has been carried out with the greatest success in Scotland for several generations, while in England not much more than a beginning has been made in it, that is, in treating them in separate departments. Dr. Rorie continues his classes for the instruction of the attendants and nurses in their duties, and prepares them for passing the examination for the Nursing Certificate of the Medico-Pyschological Association. We hope he will soon see his way to supplement this certificate by one bearing the name of his own Asylum, which dates back to 1820, and which would carry deserved weight with it. Dr. Rorie says he finds considerable difficulty in securing suitable persons for training into asylum nurses, and this complaint is a common one. We think that something more might be done as regards the salaries given to nurses, something more as to their surroundings, and, above all, it should be made certain that pensions will be given for long service. The charges for private patients range from £25 a year to £180 a year. Paupers are charged from 11s. to 12s. 6d. a week.

### THE JOINT COUNTIES ASYLUM, CARMARTHEN.

On the 1st of January, 1898, this Asylum contained 625 patients, and on the 31st of December the number had fallen to 618. There were 73 first admissions, 14 re-admissions, 26 recoveries, 4 discharged relieved, and 53 deaths. The recoveries stand at 32·91 per cent. of the admissions, and the deaths at 8·57 per cent. on the average number resident. Of the deaths during 1898, 14 were caused by cerebral and spinal diseases, 15 by consumption, 2 by pneumonia, 2 by bronchitis, and 2 by enteritis. Dr. Goodall is known to be a physician of high scientific attainments, and it would be interesting to have had his views on the cause of this disease of the intestines, which so constantly appears in the death tables of our asylums; but he does not do more than say it is common where there are defective sanitary arrangements. This may be so; but the converse does not always hold good. Of the year's admissions, 91 were driven insane by moral causes, 18 by intemperance in drink, and 28 by hereditary influence. Dr. Goodall thinks that the Inebriates Act of 1899 may extend adequate treatment "to a class of individuals who have hitherto drifted into prisons and asylums, neither of which is a fit place for the majority of these persons." As the patient must have been four times convicted of drunkenness before he can be forcibly detained in an Asylum for Inebriates, we fear that the amount of good done by that Act will not be great. The Committee say they have "great pleasure in being able to testify to the efficient management of the institution under Dr. Goodall." The average cost of maintenance per head is nearly 7s 10d. a week.

### THE ISLE OF WIGHT ASYLUM.

Here the numbers rose during 1898 from 258 to 270. There were 99 first admissions, 5 readmissions, 25 recoveries, 11 discharged relieved, and 21 deaths. The recoveries were 35·21 per cent. of the admissions, and the deaths were 8·11 per cent. of the average number resident. Of the deaths during 1898, 7 were caused by cerebral and spinal diseases, 4 by consumption, and 1 by enteric fever. Of the admissions, 27 owed their insanity to various moral causes, 13 to intemperance in drink, 22 to hereditary tendency, and in 17 the cause was unknown. As regards the case of enteric fever, Dr. Shaw traced its origin to defective drainage, and a commencement was made in reorganising the system. There has also been much trouble with the water supply, and on the date of the report water in sufficient quantity had not been found, although the boring had gone down to 330 feet below the surface. The visitors record their "appreciation of the zealous manner in which the medical superintendent has looked after the interests of the asylum." The weekly charge for maintenance is 11s 8d. per head.

### SALOP AND MONTGOMERY ASYLUM, BICTON HEATH.

In this asylum on December 31st, 1898, there were 836 patients, being an increase of one only during the year. There were 156 admissions, 54 readmissions, 72 recoveries, 33 discharged relieved, and 77 deaths. Calculated on the admissions the recoveries stand at 34·28 per cent., and calculated on the average number resident the deaths stand at 9 per cent. Of the year's deaths 28 were caused by cerebral and spinal diseases, 13 by consumption, 3 by tuberculosis, 2 by bronchitis, 3 by pneumonia, and 1 by colitis. Twenty-two of the admissions owed their insanity to hereditary influences, 17 to drink, and 7 to epilepsy. In the large proportion of 55 the disease was of unknown origin. This large number of "unknowns" makes the table comparatively useless, and we think something might have been done to reduce them by more stringent inquiries on the part of the relieving officers previous to the patients' admissions, and by the medical officers subsequent to admission. Although the numbers only increased by one during the year 1898, this was owing to the discharge of private patients to make room for paupers, a step which Dr. Strange very truly says "was greatly to be regretted, as it did away with a great boon to the class who came here as private patients, and also resulted in some financial loss to the asylum." So far Dr. Strange is right, but when he states that the step was necessary we totally disagree with him. The visitors have had for many years the power to build for private patients, and why has this been neglected? The county is the poorer for the omission, and the private patients are the worse for it. A block for 100 patients could be put up for about £10,000. The annual profits would amount to £2,000 a year or more, so that in five years the capital sum would have been repaid, and the county would have had the building for nothing. We congratulate Nurse Elizabeth Lloyd on having obtained her pension, but surely £20 a year was hardly sufficient reward for fifteen years' service in an asylum. The average weekly rate of maintenance per head was 7s. 11d.

